# Willingness to join and pay for community-based health insurance and associated determinants among urban households of Cameroon: case of Douala and Yaounde

**DOI:** 10.1016/j.heliyon.2021.e06507

**Published:** 2021-03-18

**Authors:** Rosine Wafo Cheno, William Tchabo, Jonathan Tchamy

**Affiliations:** a Department of Health Policy and Management, Jiangsu University, 301 Xuefu Road, Zhenjiang 212013, China; b Ministry of Public Health of Cameroon, Road 3038, Quartier du Lac, Yaounde, Cameroon; c University of Ngaoundere, PO Box 455, Ngaoundere, Cameroon; d Jonathan Tchamy, School of Management and Economics, Kunming University of Science and Technology, Kunming 650093, China

**Keywords:** Health prepayment scheme, Voluntary community-based health, Compulsory community-based health, Contingent valuation

## Abstract

**Background:**

The risks associated with direct health spending are high in Cameroon, where almost all household income is spent on health care. Moreover, there is no real social security in Cameroon because of the lack of a universal social protection system.

**Objectives:**

This study aimed to assess the willingness of Cameroonian urban dwellers to subscribe and the amount to pay for voluntary (VCHI) or compulsory community-based health insurance (CCHI).

**Methods:**

A cross-sectional study based on a three-stage stratified cluster random sampling design using a bidding game style based on the contingent valuation approach was performed to in the two largest urban areas of Cameroon (Douala and Yaoundé) evaluate the willingness to pay for the VCHI and CCHI.

**Results:**

The results shown that 46% of respondent were willing to join the CCHI and 41% for VCHI. Furthermore, household income, working sector, chronic disease, health priority, and family size were factors mostly associated (p < 0.001) with the willingness to join CCHI or VCHI. Increase in household income has a positive effect on household's desire for both CCHI and VCHI. While for VCHI, increase of children number resulted in an increment of the premium, contrary to the occurrence of chronic ailment which led in the decrease of the bid.

**Conclusions:**

From the results, it is clear that city dwellers in Cameroon are ready to join and pay for community health insurance. This willingness was related to their financial power which resulted in an average insurance premium of 9.03 USD and 8.17 USD, respectively for CCHI and VCHI. That bid was found to be enough for an implementation of these types of health insurance in Cameroon.

## 1 Introduction

The advancement of medical sciences and the rising standard of living have made it unacceptable for some people to suffer or die from lack of access to basic health care [1]. However, unlike developed countries, access to healthcare as a basic necessity is limited in developing countries due to the resource paucity in healthcare systems [2]. It has been well recognized by policy makers that a well-functioning heath system is imperative for the entire population to have an adequate access to health services [3]. The economic crisis in several developing countries has resulted in the reduction of healthcare budget. As consequence, looking an alternative solution to efficiently finance the health care is the most important concern of government [4]. Health insurance is typically regarded as a means to allow financial risk protection and allow the low-income family to have access to health care [5]. Social health insurance is the commonly finance scheme which has been promoted by the World Health organization to improve access to health services and guarantee universal coverage of health care delivery system [4,6]. However, middle and low-income countries rarely use this system due to the fact that a large part of the population is in the informal sector [3]. Therefore, out-of-pocket payments (OPP) and general incomes are the key sources of health care financing [4]. Thus, payments to health care providers are generally paid by individuals or families from their own wages [7]. To resolve the financial difficulties of health care for the needy, a contributory system such as community-based health insurance (CBHI) is the most probable way to attain broad-based health insurance coverage in situations where governments cannot provide direct health care support [3,8]. Cameroon is a typical case, where all most 90% of the population working in the informal sector do not pay taxes [9].

The population size of Cameroon was 17,463,836 in 2005 [10]. However, according to the UN, Cameroon's total population was estimated at 24,678,234 in 2018 [11]. Urban dwellers in Cameroon in 2018 represent over 56.4% of the national population with respectively 13.8% and 14.8% of the population living in Douala and Yaoundé, which are the two biggest and populated cities in Cameroon [11]. According to Owoundi [12], Cameroonian citizens spend about 68% of their annual income for their health care, thus leading to high risk of mortality. This behavior is more predominant in poor households which usually need to borrow or sell their property to deal with health care [3]. Among Cameroonians, solely 36.1% have access to the health center [13]. As in the majority of low-income countries, health financing is an important challenge for Cameroon. Indeed, only 6.46% of the entire population, specifically constituted by civil servants and some employees of the formal private sector are covered by a partial health insurance [14].

There are several types of health insurance that help to prevent the tragic medical events and commonly related to OPP [15]. These insurance schemes are usually categorized in compulsory, voluntary, or based on risk assessments. Among them, CBHI, which includes the compulsory and/or voluntary health schemes has been documented to provide health insurance coverage to people who cannot immediately benefit from a social or private health insurance scheme [16]. It is an alternative to private health insurance that is managed by the community-run with no risk related contributions [15]. Therefore, this type of health insurance scheme might be appropriate in Cameroon where the private health insurance seems unaffordable for the dwellers due to its high cost compared to the average income of the population [17].

Although some researchers have reported on compulsory community-based health insurance (CCHI) and voluntary community-based health insurance (VCHI) in low and middle incomes countries [16,18,19,20,21,22], there still a paucity of reports in Cameroon, especially in the urban population regarding the benefits of these schemes. Hence, this study sought to determine the factors that influence the willingness to join and pay for VCHI and CCHI, as well as to seek which type CBHI scheme might be more suitable to be implemented in an urban area in Cameroon.

## 2 Methodology

### 2.1 Sitting

In order to appreciate the socio-economic, ethnic-geographic and linguistic features of the entire country, the data were collected from a multi-region-based population. Thus, the capital of the center region (Yaounde) and littoral (Douala) region were chosen according to the economic activities, income, language, urban composition and the homogeneity of their population. Therefore, a community based cross-sectional study was performed from March to December 2018 among households of the 7 urban districts of Yaounde and the 6 urban districts of Douala, covering an estimated average population size of 3.5 million inhabitants for each city [11].

### 2.2 Ethical approval

Verbal informed consent was taken from all respondents, as well as anonymity and confidentiality at all level of the study were ensured. The study design was reviewed and approved by the ethical standards committee of the department of Health Policy and Management, School of Management, Jiangsu University. This ensured that the study does not infringe on the rights of research participants.

### 2.3 Study design

The sample size (n = 2400) of each city was calculated from Eq. (1), based on the confidence of interval (95 %, z = 1.96), standard deviation (p = 0.5), marginal error (e = 2%) and, population (N = 3500000). However, taking into account a response rate of 60%, the optimal sample size was determined to be n = 4000.

(eq. 1) $$n=\left[\left(z^{2}\times p\;\left(1-p\right)/e^{2}\right)/1+\left(z^{2}\times p\;\left(1-p\right)/e^{2}\times N\right)\right]$$

where, n is the sample size, N is the population size, e is the margin of error in percent, z is the z-score of the confidence interval, and p is the standard deviation.

A three-stage stratified cluster random sampling design was performed in order to select the participants of the survey. In the first step, the administrative capitals of each region were selected. In the second step, the administrative quarters of each urban district were chosen from each of the two regional capitals. In the third step, the line sampling technique [23] was utilized to randomly select the households. The roadmap describing the selection process of the respondents selected for the statistical analysis of the survey is described in Electronic Supplementary Material S1.

### 2.4 Survey content and interview methodology

The survey was divided into three sections in order to collect information on the variables used in the study. The information was collected based from a structured questionnaire. Section 1 collected information on socio-economic and demographic characteristics such as the age, educational background, marital status, working sector, household size, and monthly household income. Section 2 collected information on the assessment of health status of the family such as the prevalence of ailment and presence of a chronic disease. Finally, section 3 collected information on the choice and willingness to pay for health insurance plans. The interviewees were asked to express their willingness to join (WTJ) the proposed healthcare financing schemes (Electronic Supplementary Material S2). The desire to subscribe to an insurance premium was evaluated using a bidding game style based on the contingent valuation method (Electronic Supplementary Material S3).

### 2.5 Study limitations

Some factors that have been reported in the literature to influence the willingness to subscribe health insurance were not used in this study. However, the main parameters with a significant impact on the willingness to join and pay for a CBHI were selected. In addition, the concept of empowerment was not assessed in this study because the information required for this purpose was not determined during the interviews.

### 2.6 Data analysis

Descriptive statistics were employed to assess the frequency and proportion of the sociodemographic data. Furthermore, bivariate analysis was performed using Chi-squared and z-test to determine the associations between socio-economic and demographic variables with the different choices of the health insurance schemes according to Eqs. (2) and (3).

(eq. 2) $$\chi^{2}=\;\overset{k}{\underset{i=1}{\sum }}\left[{\left(O_{i}-E_{i}\right)}^{2}/E_{i}\right]$$

where $\chi^{2}$ is chi square test, O is the observed value, E is the expected value, i is the ith position in the contingency tables, and k is the degree of freedom.

(eq. 3) $$z=\left(\bar{x}-\;\text{Δ}\right)/\left(\sigma /\sqrt{n}\right)$$

where z is the z-test, $\bar{x}$ is the sample mean, Δ is the population mean (the value you are comparing it with), σ is the standard deviation of the population, and n is the number of observation (number of values in the larger set).

The variables with a *p*-value (chi-squared) less than 0.05 was selected as candidate variables for a multinomial analysis. The multinomial logit model (MLM) was performed to assess the factors that influences the respondent's WTJ the proposed health insurance policy. The probability of choosing a health insurance scheme is given by Eq. (4).

(eq. 4) $${\text{P}}_{ik}=\;\exp({\text{Υ}}_{k}X_{i}/\overset{m}{\underset{j=1}{\sum }}\exp\left({\text{Υ}}_{j}X_{i}\right)$$

where P_*ik*_ is the probability that a dweller (i) chooses a health insurance policy (k); ${\text{Υ}}_{k}$ is the effect of the socio-economic and demographic factors (X) on the probability of selecting the kth health insurance policy over the other insurance schemes, $\;X_{i}$ is the socio-economic and demographic characteristics of a dweller (i); and ${\text{Υ}}_{1},\ldots ,\;{\text{Υ}}_{j}$ are m vectors of regression coefficients.

Besides, the ordered logit model (OLM) was computed to assess the variables which influence the subscription choice among the different type of CBHI. The probability to subscribe to CCHI or VCHI schemes was determined by Eq. (5).

(eq. 5) $$P\left(Y<i\right)=\;\exp\left({\text{Υ}}_{i}-\;\beta_{j}\right)/1+\exp\left({\text{Υ}}_{i}-\;\beta_{j}\right)$$

where Y is the response variable, i is the level of an ordered category of the response variable, ${\text{Υ}}_{i}$ is the cutpoints or thresholds to depict the variations among categories, and $\beta_{j}$ is the vectors of regression coefficients for the j-th health insurance policy.

For the MLM, the relative risk ratio (RRR) was calculated to determine the probability that a respondent WTJ a CBHI would subscribe to CCHI or VCHI in relation to the likelihood to those who prefer OPP. Whilst the accuracy of the multiple logit regression was depicted by the standard error (SE) of the odds ratios. Whereas for the OLM, the odds ratios (OR) were reported as the fluctuation of odds of the result (CCHI) compared to the reference group (VCHI) per unit variation of the independent factor. In addition, marginal effects (ME) was computed to depict the impact of the change of one unit of the independent variable on the dependent variable.

Moreover, a univariate analysis using Chi-squared was carried out to analyze the variables responsible for the bid of the VCHI and CCHI. In addition to it, empirical models such as ordinary least squares linear model (OLS) and generalized linear model (GLM) (with log-link function and gamma distribution) were carried out to determine the variables that affect the amount disposed to pay for CCHI or VCHI. Therefore, the reel willingness to pay (WTP) for health insurance scheme is depicted by Eq. (6).

(eq. 6) $$WTP_{m}=\alpha +X_{m}\text{Υ}+{\text{ε}}_{m}$$

where, $WTP_{m}$ is the maximum amount willing to pay for the chosen CBHI, α is the intercept, $X_{m}$ is the vector of explanatory variables, Υ is the vector of regression coefficients, and ${\text{ε}}_{m}$ is the random error term (normal or gamma distribution).

The fitness of the model was assessed based on the likelihood ratio test (LRT), while the model selection indices Akaike information criterion (AIC) and Bayes information criterion (BIC) were calculated to determine the best model to predict the WTP according to Eqs. (7), (8), and (9).

(eq. 7) $$LRT=\;-2\;log_{e}\left[L\left({\hat{\theta }}_{nm}\right)/L_{cm}\left({\hat{\theta }}_{am}\right)\right]$$

where, LRT is the likelihood-ratio test, and $L\left(\hat{\theta }\right)$ represent the likelihood of the model (either ${\hat{\theta }}_{nm}$ for the he null model and ${\hat{\theta }}_{am}$ for the alternative model).

(eq. 8) $$AIC=2T-2\text{ln}\left(\hat{L}\right)$$

where, AIC is the Akaike information criterion, T is the number of estimated parameters by the model, and $\hat{L}$ is the maximum value of the likelihood of the model.

(eq. 9) $$BIC=\ln\left(n\right)T-2\text{ln}\left(\hat{L}\right)$$

where, BIC is the Bayes information criterion, n is the sample size, T is the number of estimated parameters by the model, and $\hat{L}$ is the maximum value of the likelihood of the model.

The descriptive and univariate analyses were carried out using IBM SPSS Statistics version 25 (IBM Corporation., Armonk, NY, USA). The multinomial logit regression, ordered logit regression, ordinary least squares linear regression and generalized linear regression model were performed with Stata/IC version 15 (Stata Corporation, College Station, TX, USA).

## 3 Result and discussion

### 3.1 Result

#### 3.1.1 Descriptive analysis

Out of the 7631 target respondents, 1331 (17.4%) were excluded from the panel (Electronic Supplementary Material S1) among them 674 declined to participate in the study, thereby leading to a response rated of 90.6% (83.9% at Yaounde, and 92.6% at Douala). As depicted in Electronic Supplementary Material S4, the majority of whom were male (71.3%), aged between 25 and 44 years (69.7%), married (76.1%), having a family size of 4–6 members (60.3%) comprising 1 to 3 children (61.8%), working in the informal sector (67.8%), having an undergraduate degree (43.0%), with a monthly household income ranging from 250000 XAF (423.03 USD) to 499999 XAF (846.05 USD) (32.5%). Furthermore, 3695 (58.6%) respondents evaluated the health status of their family as satisfactory. A total of 2057 (32.7%) of households have at least one of their relatives with chronic ailments, whilst 4952 (78.4%) respondents had contracted acute ailments requiring hospitalization 6 months before the survey. Besides, 1866 (29,6%) respondents reported spending 50000 to 149999XAF (84.61–253.81 USD) annually on health care costs.

#### 3.1.2 Bivariate analysis

Although 28.8% of respondents are knowledgeable about health insurance (Table 1), merely 87% (46 % for CCHI and 41% for VCHI) were willing to join any type of CBHI after in-deep explanation about its concept and nature, whereas 819 (13.0%) respondents preferred OPP (Table 1). However, that high willingness to subscribe for a CBHI was translated into a moderate WTP. With respect to CCHI, solely 65.5% in Yaounde and 72.7% in Douala of respondents had the WTP for the first offer of 5000 XAF (8.45 USD) (Electronic Supplementary Material S3). While those who had the WTP for this first offer with regards to VCHI accounted for 58.6% at Yaounde and 64.0% at Douala (Electronic Supplementary Material S3). Subsequently, for the next question concerning the second higher offer of 7500 XAF (12.67 USD), in Yaounde, roughly 31.6% (with respect to CCHI) and 44.9% (with respect to the VCHI) of the respondents who agreed to pay the first offer were also disposed to pay for the second higher offer of 10000 XAF (16.89 USD). While in Douala, about 34.9% (with respect to the CCHI) and 29.5% (with respect to the VCHI) of those who were willing to pay for the first offer also accepted the second higher offer. With regards to the second lower offer of 2500 XAF (4.22 USD), in Yaounde, 77.2% (with respect to scheme B) and 67.4% (with respect to scheme C) of respondents who disagreed to pay for the first offer, consented to pay the second lower offer. Whilst, in Douala, 73.5% (with respect to scheme B) and 57.0% (with respect to scheme C) of respondents who declined to register for the first offer, agreed to pay for the second lower offer. Based on the outcomes of bivariate analysis (Table 1), the predictor variables with a *p*-value lower than 0.01 such as age, education level, working sector, household income, household children, chronic illness, family size, family health status, health expenditure, marital status, acute illness, knowledge of health insurance, urban district, health priority were utilized for the multinomial analysis to assess the factors that impact on the choice of health insurance scheme.

**Table 1:** Distribution of respondents’ choices of the different types of health insurance schemes.

Variables	Choice of health insurance scheme			Prob > chi^2^
	A	B	C	
Overall	819(13.0%)	2898(46.0%)	2583(41.0%)	
Urban district				∗∗
Yaounde	378^a^(12.0%)	1512^b^(48.0%)	1260^a^(40.0%)	
Douala	441^a^(14.0%)	1386^b^(44.0%)	1323^a^(42.0%)	
Marital status				∗∗∗
Single	174^a^(28.3%)	149^b^(24.3%)	291^c^(47.4%)	
Married	391^a^(8.2%)	2464^b^(51.4%)	1939^c^(40.4%)	
Divorced	144^a^(29.3%)	185^b^(37.6%)	163^b^(33.1%)	
Widowed	110^a^(27.5%)	100^b^(25.0%)	190^C^(47.5%)	
Working sector				∗∗∗
Public	214^a^(19.5%)	587^b^(53.5%)	297^C^(27.0%)	
Private	15^a^(1.8%)	732^b^(88.9%)	76^a^(9.2%)	
Informal	540^a^(12.6%)	1544^b^(36.1%)	2190^c^(51.2%)	
Retired	50^a^(47.6%)	35^b^(33.3%)	20^b^(19.0%)	
Education level				∗∗∗
None	9^a^(8.4%)	84^b^(78.5%)	14^a^(13.1%)	
Primary	32^a^(7.4%)	262^b^(60.2%)	141^a^(32.4%)	
Secondary	62^a^(3.1%)	822^b^(40.6%)	1141^c^(56.3%)	
Undergraduate	327^a^(12.1%)	1403^b^ (51.8%)	980^a^(36.2%)	
Postgraduate	389^a^(38.0%)	327^b^(32.0%)	307^b^(30.0%)	
Age				∗∗∗
18-24	405^a^(68.8%)	73^b^(12.4%)	111^c^(18.8%)	
25-44	273^a^(6.2%)	2423^b^(55.2%)	1695^c^ (38.6%)	
45-64	126^a^(10.5%)	356^b^(29.7%)	715^c^(59.7%)	
≥ 65	15^a,b^(12.2%)	46^b^(37.4%)	62^a^(50.4%)	
Family size				∗∗∗
1-3	585^a^(28.4%)	495^b^(24.1%)	977^c^(47.5%)	
4-6	199^a^(5.2%)	2091^b^(55.0%)	1509^c^(39.7%)	
≥7	35^a^(7.9%)	312^b^(70.3%)	97^a^(21.8%)	
Household children				∗∗∗
0	622^a^(30.5%)	495^b^(24.3%)	920^c^(45.2%)	
1-3	179^a^(4.6%)	2091^b^(53.7%)	1622^c^(41.7%)	
≥4	18^a^(4.9%)	312^b^(84.1%)	41^a^(11.1%)	
Household income (XAF)^Ɣ^				∗∗∗
< 50000	18^a^(10.4%)	129^b^(74.6%)	26^c^(15.0%)	
50000–149999	112^a^(5.7%)	1542^b^(78.3%)	316^a^(16.0%)	
150000–249999	206^a^(10.9%)	668^a^(35.5%)	1009^b^(53.6%)	
250000–499999	381^a^(18.6%)	514^b^(25.1%)	1152^a^(56.3%)	
≥500000	102^a^(44.9%)	45^b^(19.8%)	80^c^(35.2%)	
Health expenditure (XAF)^Ɣ^				∗∗∗
None	511^a^(37.7%)	312^b^(23.0%)	532^c^(39.3%)	
< 50000	161^a^(9.1%)	888^b^(50.3%)	718^c^(40.6%)	
50000–149999	71^a^(3.8%)	808^b^(143.3%)	987^c^(52.9%)	
150000–249999	38^a^(5.5%)	401^b^(58.4%)	248^c^(36.1%)	
250000–499999	24^a^(5.4%)	348^b^(77.7%)	76^a^(17.0%)	
≥500000	14^a^(7.9%)	141^b^(79.7%)	22^c^(12.4%)	
Chronic illness				∗∗∗
No	761^a^(17.9%)	1494^b^(35.2%)	1988^c^(46.9%)	
Yes	58^a^(2.8%)	1404^b^(68.3%)	595^c^(28.9%)	
Family health status				∗∗∗
Bad	56^a^(5.6%)	672^b^(67.6%)	266^c^(26.8%)	
Passable	154^a^(4.2%)	1896^b^(51.4%)	1642^b^(44.5%)	
Good	609^a^(37.7%)	330^b^(20.4%)	675^c^(41.8%)	
Acute illness				∗∗∗
No	517^a^(38.1%)	309^b^(22.8%)	532^c^(39.2%)	
Yes	302^a^(6.1%)	2589^b^(52.4%)	2051^c^(41.5%)	
Health priority				∗∗∗
Low	50^a^(52.6%)	14^b^(14.7%)	31^c^(32.6%)	
Medium	357^a^(48.1%)	207^b^(27.9%)	178^b^(24.0%)	
Hight	412^a^(7.5%)	2677^b^(49.0%)	2374^b^(43.5%)	
Be aware of health insurance				∗∗∗
No	430^a^(9.6%)	2082^b^(46.4%)	1973^c^(44.0%)	
Yes	389^a^(21.4%)	816^b^(45.0%)	610^c^(33.6%)	

∗, ∗∗, and ∗∗∗ indicate *p* < 0.05, *p* < 0.01, *p* < 0.001 significant levels, and non-significant, respectively (Chi^2^ test).

Each subscript letter denotes a subset of variables whose column proportions do not differ significantly from each other at p < 0.05 (Z-test).

A (out-of-pocket), B (Compulsory scheme) and C (voluntary scheme).

^Ɣ^1 USD = 590.98 XAF.

#### 3.1.3 Regression analysis

The outcomes of the triple bounded MLM highlighting the significant impact of socio-economic and demographic factors on the dweller's probability to join health policy schemes is presented in Table 2. Models I, II and III comprised the same covariates variables excluding the occurrence of acute illness, awareness of health insurance, urban district and health priority for models 2 and 3, as well as the family size, family health status, Health expenditure, and Marital status for model 3. On the basis of the LRT, AIC, and BIC, the model1 was found to be the most accurate to explain the factors affecting the WTJ for the different health insurance scheme. The model I outcomes (Table 2) revealed that except the occurrence of chronic illness in the households and the family size with respect to VCHI, all the other factors significantly influence the WTJ CBHI as compare to the OPP. The odds of respondents who had a high priority of their health to choose CCHI instead of OPP were 6.52 time higher than those who gave low priority to their health. Besides, the aged respondents were 6.44 more willing to subscribe for the scheme VCHI than OPP. Interestingly, the urban district was noted to be the factor that significantly (*p* < 0.05 for VCHI, and *p* < 0.01 for CCHI) influencing less to the WTJ a CBHI. Furthermore, based on the ME outcomes (Table 3), the age (with respect to scheme C) education level, working sector, house income, house children (with respect to scheme B), chronic illness, family size, and the awareness of health insurance (with respect to scheme C) were found to be the main predictive variables in the decision of dwellers to enroll in CBHI. Positive ME was related to the WTJ a CBHI converse to negative ME which indicated a disagreement to join the type CBHI. Noticeably, categorial predictive variables were noted to have converse effect on the WTJ the CCHI and VCHI (Table 3).

**Table 2:** Multinomial logit regression model of health insurance schemes.

Model		I		II		III	
Independent variables		Scheme B	Scheme C	Scheme B	Scheme C	Scheme B	Scheme C
Age	RRR	5.10∗∗∗	6.44∗∗∗	6.31∗∗∗	7.50∗∗∗	5.98∗∗∗	4.39∗∗∗
	SE	0.70	0.78	0.81	0.84	0.63	0.40
Education level	RRR	0.53∗∗∗	0.41∗∗∗	0.54∗∗∗	0.39∗∗∗	0.52∗∗∗	0.40∗∗∗
	SE	0.05	0.03	0.05	0.03	0.04	0.03
Working sector	RRR	0.24∗∗∗	0.76∗∗∗	0.28∗∗∗	0.92	0.35∗∗∗	0.99
	SE	0.02	0.06	0.02	0.06	0.02	0.06
Household income	RRR	0.10∗∗∗	0.54∗∗∗	0.12∗∗∗	0.68∗∗∗	0.14∗∗∗	0.63∗∗∗
	SE	0.01	0.04	0.01	0.05	0.01	0.04
Household children	RRR	3.94∗∗∗	2.73∗∗∗	5.55∗∗∗	3.80∗∗∗	14.74∗∗∗	4.69∗∗
	SE	0.62	0.40	0.81	0.51	1.69	0.48
Chronic illness	RRR	2.96∗∗∗	1.01	2.48∗∗∗	0.83	5.79∗∗∗	1.63∗∗∗
	SE	0.60	0.20	0.45	0.15	0.95	0.26
Family size	RRR	5.75∗∗∗	1.14	4.94∗∗∗	0.99		
	SE	0.86	0.16	0.70	0.13		
Family health status	RRR	0.31∗∗∗	0.35∗∗∗	0.27∗∗∗	0.32∗∗∗		
	SE	0.05	0.05	0.04	0.04		
Health expenditure	RRR	0.46∗∗∗	0.47∗∗∗	0.83∗	0.85∗		
	SE	0.04	0.04	0.06	0.06		
Marital status	RRR	0.25∗∗∗	0.26∗∗∗	0.31∗∗∗	0.31∗∗∗		
	SE	0.02	0.02	0.03	0.02		
Acute illness	RRR	4.50∗∗∗	4.71∗∗∗				
	SE	0.96	0.90				
Be aware of health insurance	RRR	0.57∗∗∗	0.43∗∗∗				
	SE	0.08	0.05				
Urban district	RRR	0.69∗∗	0.79∗				
	SE	0.09	0.09				
Health priority	RRR	6.52∗∗∗	5.35∗∗∗				
	SE	0.99	0.70				
_cons	RRR	762.92∗∗∗	47.96∗∗∗	4804.61∗∗∗	247.28∗∗∗	150.09∗∗∗	15.36∗∗∗
	SE	368.78	21.67	2164.38	103.22	48.01	4.60
	Prob > chi^2^	0.000		0.000		0.000	
	Pseudo R^2^	0.44		0.40		0.34	
	AIC	7084.65		7455.29		8201.60	
	BIC	7287.09		7603.75		8296.08	
	LR Test model I vs model II (LR chi^2^ = 386.64, *p* = 0.000)						
	LR Test model I vs model III (LR chi^2^ = 1148.96, *p* = 0.000)						
	LR Test model II vs model III (LR chi^2^ = 762.31, *p* = 0.000)						

∗, ∗∗, and ∗∗∗ indicate *p* < 0.05, *p* < 0.01, *p* < 0.001 significant levels, and non-significant, respectively (Z-test).

B (Compulsory scheme) and C (voluntary scheme), RRR (relative risk ratios), SE (standard error), AIC (Akaike's information criterion), and BIC (Bayesian information criterion).

Note: Out-of-pocket was select as the base outcome.

**Table 3:** Marginal effects of the multinomial logit regression model of health insurance schemes.

Model	I		II		III	
Independent variables	Scheme B	Scheme C	Scheme B	Scheme C	Scheme B	Scheme C
Age	-0.03	0.08∗∗∗	-0.01	0.08∗∗∗	0.11∗∗∗	-0.03∗
Education level	0.05∗∗∗	-0.07∗∗∗	0.06∗∗∗	-0.09∗∗∗	0.05∗∗∗	-0.08∗∗∗
Working sector	-0.29∗∗∗	0.27∗∗∗	-0.30∗∗∗	0.27∗∗∗	-0.26∗∗∗	0.24∗∗∗
Household income	-0.44∗∗∗	0.40∗∗∗	-0.43∗∗∗	0.38∗∗∗	-0.38∗∗∗	0.33∗∗∗
Household children	0.10∗∗∗	-0.07∗∗	0.12∗∗∗	-0.06∗∗	0.32∗∗∗	-0.22∗∗∗
Chronic illness	0.26∗∗∗	-0.25∗∗∗	0.26∗∗∗	-0.25∗∗∗	0.32∗∗∗	-0.27∗∗∗
Family size	0.40∗∗∗	-0.38∗∗∗	0.40∗∗∗	-0.37∗∗∗		
Family health status	-0.05∗	0.02	-0.07∗∗	0.02		
Health expenditure	-0.01	-0.01	-0.01	0.00		
Marital status	-0.02	-0.01	-0.02	-0.02		
Acute illness	0.02	0.05				
Be aware of health insurance	0.06∗	-0.08∗∗∗				
Urban district	-0.04∗	0.03				
Health priority	0.07∗	-0.02				

∗, ∗∗, and ∗∗∗ indicate *p* < 0.05, *p* < 0.01, *p* < 0.001 significant levels, and non-significant, respectively (Z-test).

B (Compulsory scheme) and C (voluntary scheme).

Note: Out-of-pocket was select as the base outcome.

The results of the OLM depicting the effects of the explanatory variables on the respondents who are willing to adhere to a health insurance policy are tabulated in Table 4 & Electronic Supplementary Material S5. The model IV, including all the independent variables, was demonstrated to be the most appropriate to represent the actual relationship between the willingness to adhere CCHI or VCHI and the socio-economic and demographic characteristics of the population. As can show in Table 4, the estimated OR and ME indicated that the education, working sector, household income, chronic illness and family size were the most discriminative variables affecting the choice of the type of CBHI. Thus, based on the partial effect (Electronic Supplementary Material S5) of the most significant variables related to the willingness to adhere to CCHI or VCHI, the increase in household income and membership of the informal sector led to an increase in the probability of subscribing to VCHI. While, the presence of a chronic disease, an increase in the health priority, as well as in the size of the family resulted to an increase in the likelihood of adhering to CCHI.

**Table 4:** Ordered logit regression model of the choice of the community-based health insurance.

Model	IV			V		
Independent variables	OR	SE	ME	OR	SE	ME
Age	1.29∗∗	0.12	-0.06∗∗	1.21∗	0.10	-0.05∗
Education level	0.75∗∗∗	0.04	0.07∗∗∗	0.72∗∗∗	0.04	0.08∗∗∗
Working sector	3.42∗∗∗	0.20	-0.30∗∗∗	3.53∗∗∗	0.20	-0.31∗∗∗
Household income	5.89∗∗∗	0.32	-0.44∗∗∗	5.77∗∗∗	0.31	-0.43∗∗∗
Household children	0.75∗∗	0.07	0.07∗∗	0.73∗	0.07	0.08∗∗
Chronic illness	0.33∗∗∗	0.03	0.26∗∗∗	0.31∗∗∗	0.03	0.27∗∗∗
Family size	0.18∗∗∗	0.02	0.42∗∗∗	0.18∗∗∗	0.02	0.42∗∗∗
Health expenditure	1.00	0.04	0.00	0.95	0.04	0.01
Family health status	1.14	0.11	-0.03			
Health priority	0.77∗	0.09	0.07∗			
Marital status	0.99	0.07	0.00			
Urban District	1.12	0.08	-0.03			
Be aware of health insurance	0.80∗	0.08	0.05∗			
/cut	2.76	0.31		2.84	0.20	
Prob > chi^2^	0.000			0.000		
Pseudo R^2^	0.38			0.38		
AIC	4712.985			4719.78		
BIC	4805.511			4779.261		
	LR Test model I vs model II (LR chi^2^ = 16.19, *p* = 0.005)					

∗, ∗∗, and ∗∗∗ indicate *p* < 0.05, *p* < 0.01, *p* < 0.001 significant levels, and non-significant, respectively (Z-test).

OR (Odd ratio), SE (standard error), ME (Marginal effect), AIC (Akaike's information criterion), and BIC (Bayesian information criterion).

Note: Voluntary community-based health insurance was select as the base outcome.

The univariate analysis revealed that the average amount of the WTP for a CBHI in Cameroon was 5094.69 XAF (8.62 USD) per month (SD = 2730.85 XAF; median = 5000 XAF). As depicted in Electronic Supplementary Material S6, respondents were disposed to pay 5334.37 XAF (9.03 USD) as monthly subscription (SD = 2674.20 XAF; median = 5000 XAF) for those who chose CCHI and 4825.78 XAF (8.17 USD) with regards to the VCHI (SD = 2768.96 XAF; median = 5000 XAF). Furthermore, the premium (tariff) was noted to be highly influenced (*p* < 0.001) by all the covariates factors (Electronic Supplementary Material S6). Hence, it was found that the maximum WTP for CCHI or VCHI was highest for female, the inhabitants of Douala, the married persons, the postgraduate degree holders, those who have a high priority for their health, those aged from 25 to 64 years, those with a large family, as well as a high family income and high health expenditures.

The outcomes of the OLS and GLM models employed to assess the relationship between the population's socio-economic and demographic characteristics and the price that Cameroonian dwellers are willing to pay for a CBHI are presented in Table 5. Owing to the AIC and BIC computed using the estat ic function based on the likelihood, the OLS was found to be the most accurate to identify the population's features mainly responsible for the premium of CCHI or VCHI. Moreover, the significant predictive variables for CCHI and VCHI were found to be age (only for CCHI), education level (only for VCHI), household children (only for VCHI), household income, chronic illness (only for VCHI), family health status (only for VCHI), health priority (only for VCHI), urban district, and awareness of health insurance (only for VCHI). Furthermore, with regards to the CCHI and VCHI, the household income was noted to be the factor weighing the more for the bid decision. Hence, based on the most significant (*p* < 0.001) partial effects (Electronic Supplementary Material S7), an increase in household income led to an increase of bid for both CCHI and VCHI. Additionally, dwellers from Douala were more like to pay high premium than those of Yaoundé. Besides, with regards of VCHI, the increase of children number in household resulted in an increment of the bid. While, the presence of chronic ailment had a negative impact on the offer for VCHI.

**Table 5:** Ordinary least squares and generalized linear model of the premium of community-based health insurance.

Model	Scheme B				Scheme C			
	OLS		GLM		OLS		GLM	
Independent variables	Coef.	SE	Coef.	SE	Coef.	SE	Coef.	SE
Age	-0.06∗	0.03	-0.01	0.01	0.03	0.03	0.02	0.01
Education level	-0.01	0.02	0.01	0.01	0.09∗∗∗	0.02	0.03∗∗∗	0.01
Working sector	0.00	0.02	-0.01	0.01	-0.03	0.02	-0.02∗	0.01
Household children	0.03	0.03	0.03∗∗	0.01	0.15∗∗∗	0.03	0.05∗∗∗	0.01
Household income	1.02∗∗∗	0.02	0.32∗∗∗	0.01	1.21∗∗∗	0.02	0.50∗∗∗	0.01
Chronic illness	-0.02	0.03	0.00	0.01	-0.18∗∗∗	0.04	-0.06∗∗∗	0.02
Health expenditure	0.03	0.01	0.00	0.01	-0.03	0.02	-0.01	0.01
Family health status	0.04	0.03	0.01	0.01	-0.10∗∗	0.04	-0.02	0.01
Health priority	0.03	0.05	0.05∗∗	0.02	-0.21∗∗∗	0.05	-0.02	0.02
Urban District	0.19∗∗∗	0.03	0.06∗∗∗	0.01	0.12∗∗∗	0.03	0.04∗∗∗	0.01
Be aware of health insurance	0.01	0.03	0.00	0.01	-0.09∗	0.04	-0.05∗∗∗	0.01
_cons	1.33∗∗∗	0.11	0.40∗∗∗	0.04	0.25	0.13	-0.25∗∗∗	0.05
AIC	5875.52		12140.34		5607.60		10283.30	
BIC	5947.18		12212.00		5677.88		10353.58	

∗, ∗∗, and ∗∗∗ indicate *p* < 0.05, *p* < 0.01, *p* < 0.001 significant levels, and non-significant, respectively (Z-test).

B (Compulsory scheme) and C (voluntary scheme), OLS (Ordinary least square model), GLM (generalized linear model), OR (relative risk ratios), SE (standard error), AIC (Akaike's information criterion), and BIC (Bayesian information criterion).

### 3.2 Discussion

Cameroon is a developing country with lowest performance in health risk in sub-Saharan Africa leading to a high out of pocket healthcare payment accounting to 94.6% of total household health expenditure [24]. The effective implementation of a health insurance system (either communal or private, voluntary or compulsory) can be the beginning of a solution to the problem of universal health coverage.

In this study, it was revealed that knowledge about health insurance by the inhabitants was low. This low rate of knowledge (28.8%) depicted in Electronic Supplementary Material S4 might be attributed to the lack of public advocacy by the media and awareness campaigns [25,26,27]. Similar findings had previously reported in Cameroon [25,27], Nigeria [28], India [29] and Myanmar [26]. However, after a deep explanation about the concept of health insurance, most of the (87%) respondents were willing to subscribe to CBHI (Table 1). This high level of approval was in line with similar studies performed in Nigeria [30], Malaysia [15], Cameroon [25,27] and Myanmar [26]. According to Noubiap et al. [25], people in low-income countries are more motivated to subscribe to a health insurance policy if they are well informed of their benefits. With respect to the type of CBHI, 46% (48% in Yaounde and 44% in Douala) of inhabitants were WTJ the CCHI conversely to 41% (42% in Yaounde and 40% in Douala) of dweller which agree to subscribe the VCHI. There is a paucity in scientific literature of reports comparing the preferences between health insurance plans. From the authors knowledge only few studies [15,22,31] were conducted on the preference between VCHI and CCHI. Our findings were similar to that found in Vietnam [22] where the inhabitants were more WTJ the CCHI than VCHI contrary to Malaysia [15] and Ethiopia [31] where most of citizens agree to subscribe for the VCHI. According to Shafie, Hassali [15], this discrepancy with Malaysia [15] may be ascribe to the fact that CCHI is more attractive for countries with low income like Cameroon or Vietnam, since the bid is chiefly related to the household income. This statement is in agreement with our findings, which revealed that an increase in household income increases the probability to join the VCHI as tabulated in Table 4 & Electronic Supplementary Material S5. Hence, the converse findings observed with that of Ethiopia [31] which is also a low-income country might be ascribed to difference in benefits package between the proposed voluntary schemes. As reported by previous authors [8,21], the choice of the type of health insurance significantly relies on the package (number of beneficiaries, quality of health care, limited disease coverage,...) of the proposed insurance scheme. Surprisingly, all the socio-economic and demographic factors investigated in this study were found to significantly impact of the choice of the type CBHI. These determinants had been reported to be significantly influenced by the choice of CBHI in others countries [8,26,29,30,32,33]. However, the chronic illness and family size did not show any significant association with the preference of VCHI in the Multinomial logit regression analysis albeit it did in the univariate logit analysis. This dissimilarity might be attributed to differences in the socio-economic status related to the study areas.

The implementation of a health insurance plan does not only depend on the WTJ, but especially on the WTP. It is interesting to note that all respondents in this study who wanted to subscribe to health insurance also agreed to pay for it. As stated by Dror et al. [32] a good knowledge and understanding of the CBHI's principles increases the WTP for it. Hence, the mean amount to pay monthly per households for CCHI was 5334.37 XAF (9.03 USD) and for VCHI was 4825.78 XAF (8.17 USD). The outcomes of the present study, were in line with the average amount of willingness to pay for VCHI in urban areas of other low-income countries like 4.37 in Nigeria [34], 6.60 USD in Namibia [1] and 7.34 USD in Sierra Leone [35]. There is limited information in the literature regarding the WTP for CCHI. However, Entele, Emodi [31] found an average WTP of 1.61 USD monthly per households for CCHI in a rural area of Ethiopia. This discrepancy might be due to the better households income, education level, and awareness about health insurance of urban dwellers than those leaving in rural area [35]. Although several studies have determined the average amount of willingness to pay for CBHI, there is lack of information about the feasibility of implementing these insurances based on the amounts proposed by the respondents. The average household size was reported to be 4 in Douala et Yaounde [36]. In addition, as stated by Bizolé et al. [37], the annual health spending in Cameroon was 6.8 USD per capita in 2018. Assuming that the probability of to attend hospital is 0.5, and taking into account the willingness to pay CCHI per capita is 2.26 USD per month. Our results have shown that it will take a subsidy of around 66.8% to cover the health needs of 80.0% of the community if 40% of the inhabitants pay the average offer. According to Asenso-Okyere et al. [38], in developing countries less than 60% of the population is able to pay health insurance costs. By the way, Mao et al. [39] have reported that in China, the government had subsidized health insurance to compensate for the default of the needy to avoid the impact of financial disasters related to health [40]. On the other hands, concerning VCHI our results demonstrated that the health care costs of the entire community could be covered if 60.1% of the habitants pay for the average premium found based on a probability of 0.5 of being sick. Our finding was found to be higher than that of Malaysia by Shafie, Hassali [15]. This discrepancy might be attributed to the difference in the GNI per capita between the two countries. As reported by previous authors [27,41,42], income in developing countries is one of the most significant parameter that affect the willingness and the amount to pay for health insurance. This statement was consistent with our results, which portrayed that increase in income led to high amount to pay for CBHI (Electronic Supplementary Material S7).

This report provides useful information on the preferences of the Cameroonian population for community-based health insurance. The results show that in a low-middle-income country without universal access to health care, such as Cameroon, the population of urban cities tends to prefer CCHI over VCHI. As above portrayed CCHI is more appealing to Cameroonians, probably because it does not take into account the size and prevalence of disease in the family. This attitude is reflected in our study, in which large families with many children and those with a chronic disease occurrence offered a higher amount of insurance. Hence, policymakers could use these outcomes to formulate an optimal combination of health care funding in Cameroon and consider CCHI as the most suitable type CBHI for urban dwellers as protection against health-related financial disasters. However, it implies that for an effective implementation of this type of health insurance, it will require the contribution of exogenous factors, in the form of external government support and the ability to increase the number of community subscriptions. Thus, in the absence of sufficient memberships, the pool of funds may not be sufficient to cover the medical benefits of the insured people and the survival of insurance will rest only on government funding. On the other hand, albeit, CCHI is attractive to most inhabitants, an increasing attention has been paid to the VCHI as a probable alternative of the health care coverage. Nonetheless, it is important to highlight that the effectiveness of CBHI do not only depends on the parameters assessed but also on some others factors which are not investigated in this study. Therefore, since the average annual amount per person for the CBHI in Yaounde and Douala (27.1 USD for CCHI and 24.5 USD for VCHI) was less than that of health expenditure per capita per year (81.6 USD), hence the subscription to the CBHI is more attractive for the citizens of an impoverished urban community that does not have easy access to public health services. However, even though this study is representative and valid for the urban population, we can expect a lower estimate for the entire Cameroonian urban population, given that Douala and Yaounde are the two cities with the highest economic capital of Cameroon. In addition, as stated by Shafie, Hassali [15], the WTP is not necessarily equal to the premium because it only represents the moral and financial acceptability of the participant.

## 4 Conclusion

Overall, this study found that community awareness of health insurance and its benefits needs to be increased. Our results showed that compulsory and voluntary health insurance could be accepted in urban areas of Cameroon as an alternative means of financing health care. The willingness to join the community-based health insurance had been influenced by socio-economic factors such as age, education level, working sector, marital status and health expenditure. Hence, for an effective implementation of these types of insurance, the decision on the amount to be paid must take into account the household's income, the educational level, the size and the health status of the families. In addition, the viability of compulsory community-based health insurance will require political will and a commitment from the government to subsidize this program to address the deficit caused by members who cannot pay. On the other hand, the increased awareness within the community on the importance of insurance in the face of the risk associated with direct payment of health care could lead the population more to take out for voluntary insurance and therefore the sustainability depends on the number of people willing to pay.

Although, the main goal of this research has been achieved, there is however, room for further studies on the implementation of health insurance in Cameroon, especially, the concept of empowerment of health insurance which has to be assess.

## Declarations

### Author contribution statement

Rosine Wafo Cheno: Conceived and designed the experiments; Analyzed and interpreted the data; Wrote the paper.

William Tchabo: Analyzed and interpreted the data; Wrote the paper.

Jonathan Tchamy: Analyzed and interpreted the data.

### Funding statement

This research did not receive any specific grant from funding agencies in the public, commercial, or not-for-profit sectors.

### Data availability statement

Data will be made available on request.

### Declaration of interests statement

The authors declare no conflict of interest.

### Additional information

No additional information is available for this paper.

## Appendix A Supplementary data

Electronic_Supplementary_Material_V2 Electronic_Supplementary_Material_V2
