# Supplementary material for: Willingness to join and pay for community-based health insurance and associated determinants among urban households of Cameroon: case of Douala and Yaounde
Source: Heliyon. 2021 Mar 18;7(3):e06507. doi: 10.1016/j.heliyon.2021.e06507 (PMC8010406; doi:10.1016/j.heliyon.2021.e06507)
Supplement: Electronic_Supplementary_Material_V2 [file mmc1.docx]

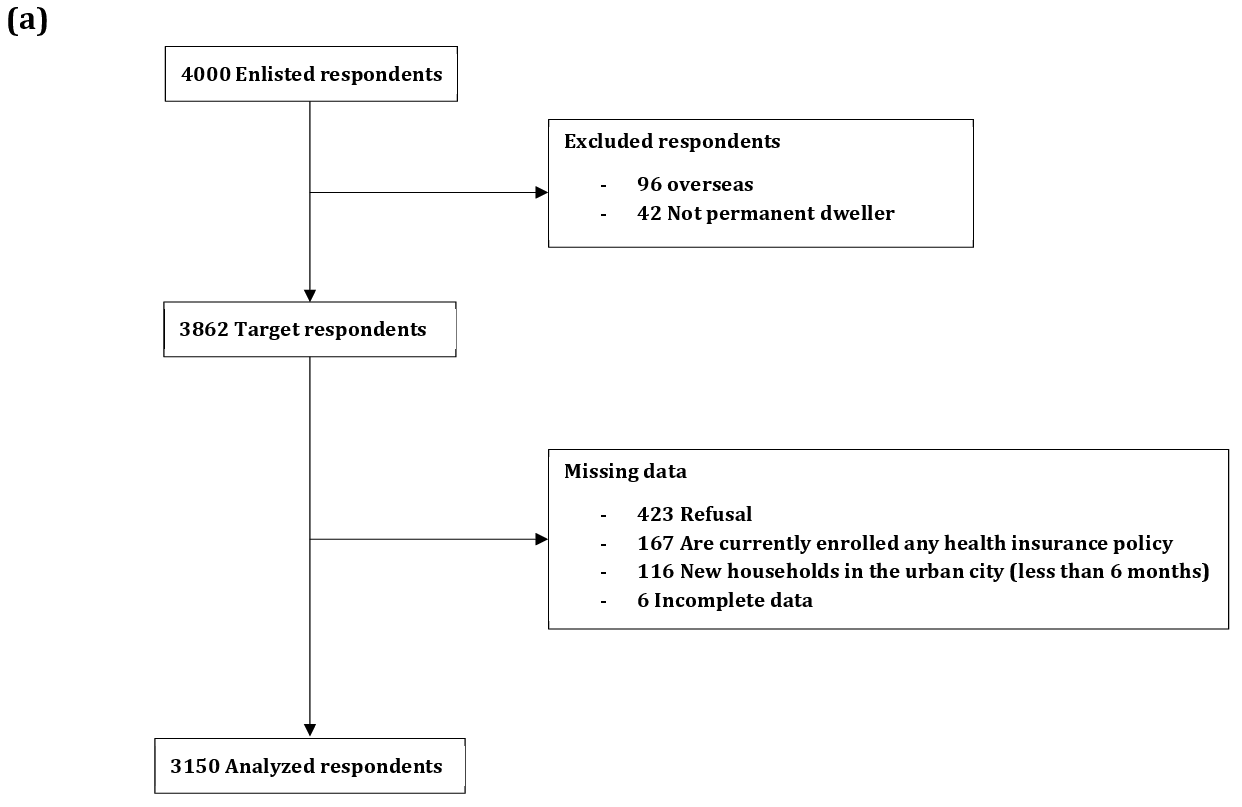


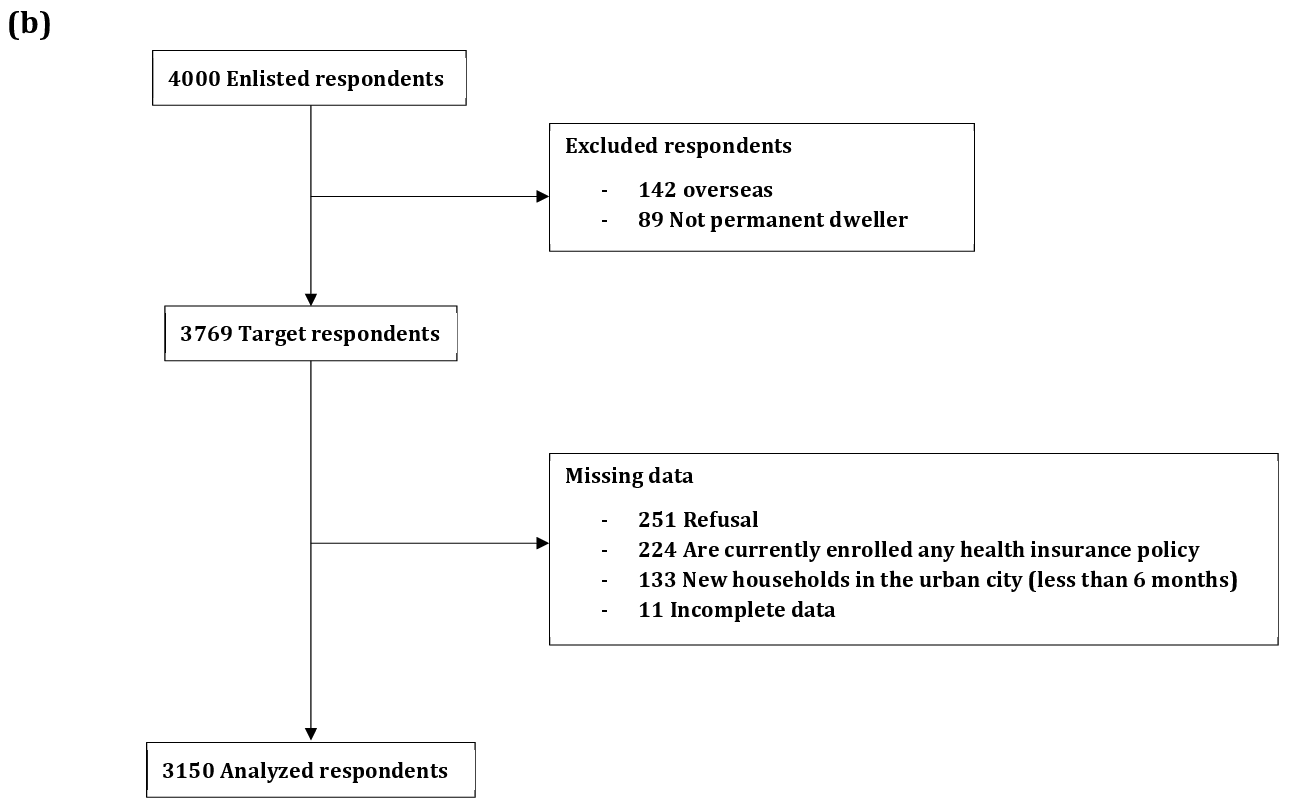


**Supplementary data S1:** Flow chart of respondent’s selection

a-Yaounde, and b-Douala

**Electronic Supplementary Material S2:** Proposed health financial scheme scenarios

| [Payment method of healthcare service](https://patents.google.com/patent/US20020010594A1/en) | Hypothetical scenarios | |
| --- | --- | --- |
| OPP | A | The cost of medical care such as medical consultation, paramedical exam, hospitalization, and medication prescription at the district hospitals, district medical centers, and integrated health centers are entirely borne by households. Medical care must (without exemption) be pay before to be done. Each medical act has it one's cost. |
| CCHI | B | A monthly premium is obliged (**COMPULSORY**) to be pay by all households without exemption to a district health care fund. The cost of the insurance will be based on household income. Hence, the higher the income level, the higher the fees. Thus, medical services like medical consultations, laboratory tests, prescribed drugs, medical supplies are utterly free for all household members at the district hospitals/district medical centers/integrated health centers. If hospitalization is needed, the insurance will cover the relative expenses for up to 15 days (per family annually). |
| VCHI | C | A monthly **VOLUNTARILY** premium may be paid to a district health care fund (community-based health insurance) by each household. The apportionment of the insurance cost will be based on the number and the ages of family members. Hence, high prices will be attributed to Children (under five) and elderly (above sixty-five) due to the fact that they are more likely to attend. hospital. Thereby, free medical cares at the district hospitals/district medical centers/integrated health centers and prescribed drugs will be given to everybody having paid the insurance fees. If hospitalization is needed, the insurance will cover the daily relative expenses. |

OPP (out-of-pocket), CCHI (compulsory community-based health insurance), and VCHI (voluntary community-based health insurance).

Adapted from [Lofgren, Thanh et al. (2008)](#_ENREF_1).


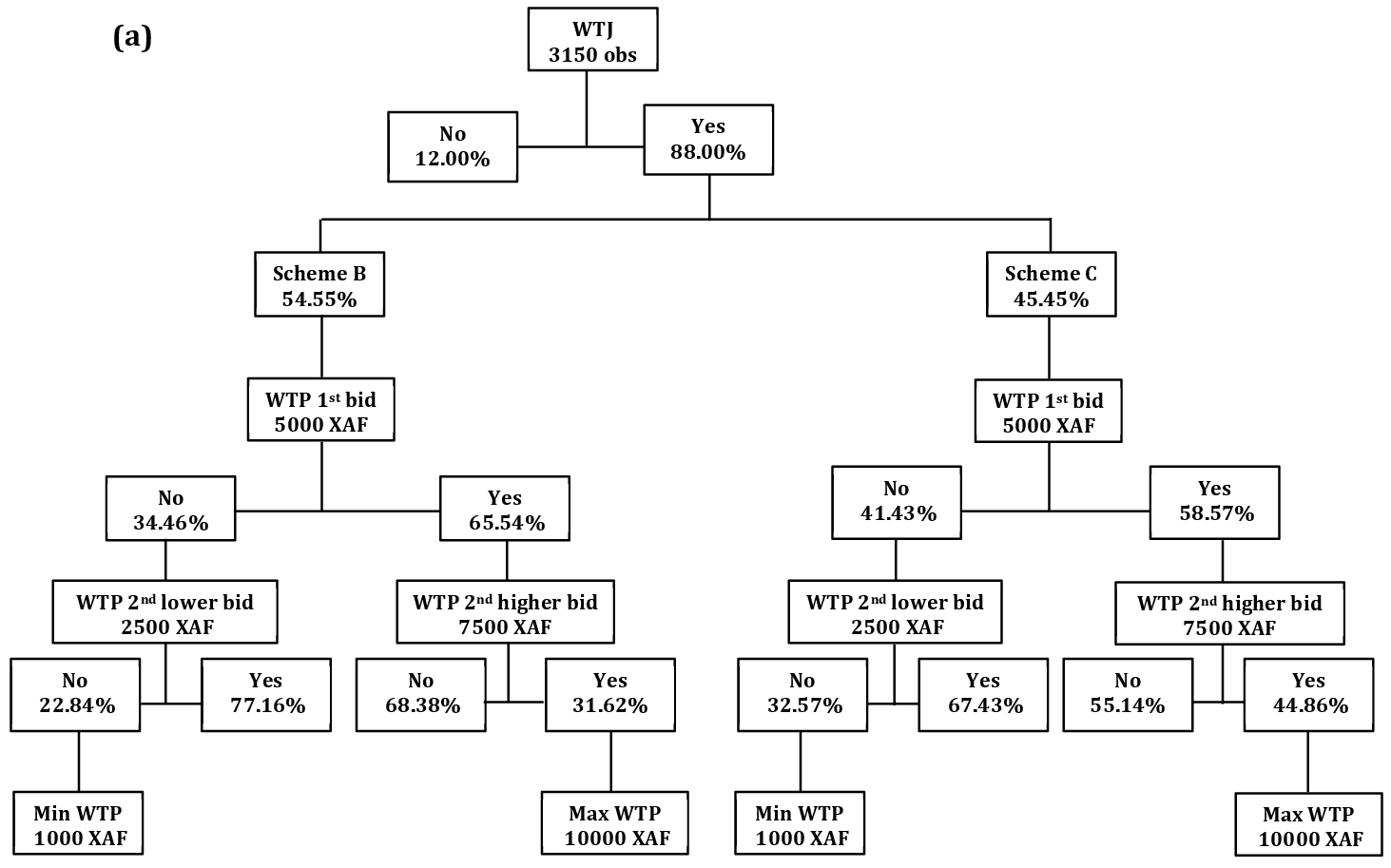


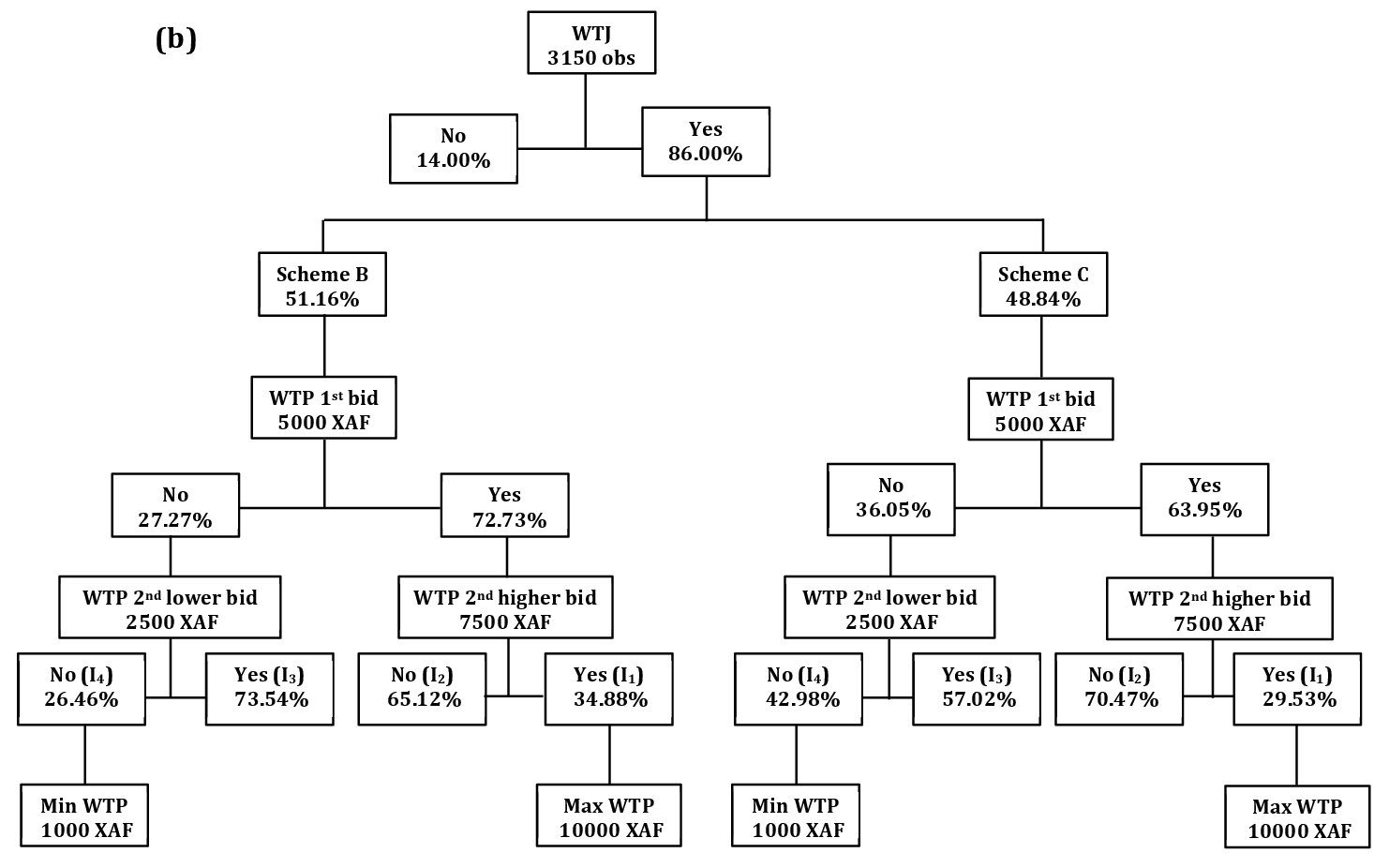


**Supplementary data S3:** Flow chart summary of the willingness to pay for community health assurance

a-Yaounde, and b-Douala

I1: if the respondent agrees to the first and second higher bids;

I2: if the respondent agrees to the first bid and disagrees to the second higher bids

I3: if the respondent disagrees to the first bid and agrees to the second lower bids

I4: if the respondent disagrees to the first and second lower bids.

**Supplementary data S4:** Sociodemographic characteristics

| Variables |  | n (%) |
| --- | --- | --- |
| Urban district | Yaounde | 3150 (50.0) |
|  | Douala | 3150 (50.0) |
| Gender | Male | 4491 (71.3) |
|  | Female | 1809 (28.7) |
| Marital status | Single | 614 (9.7) |
|  | Married^Ѣ^ | 4794 (76.1) |
|  | Divorced | 492 (7.8) |
|  | Widowed | 400(6.3) |
| Working sector | Public | 1098 (17.4) |
|  | Private | 823 (13.1) |
|  | Informal^ф^ | 4274 (67.8) |
|  | Retired | 105 (1.7) |
| Education level | None | 107 (1.7) |
|  | Primary | 435 (6.9) |
|  | Secondary | 2025(32.1) |
|  | Undergraduate | 2710 (43.0) |
|  | Postgraduate | 1023 (16.2) |
| Family health status | Bad | 994 (15.8) |
|  | Passable | 3692 (58.6) |
|  | Good | 1614 (25.6) |
| Chronic illness | No | 4343 (67.3) |
|  | Yes | 2057 (32.7) |
| Age | 18-24 | 589 (9.3) |
|  | 25-44 | 4391 (69.7) |
|  | 45-64 | 1197 (19.0) |
|  | ≥65 | 123 (2.0) |
| Be aware of health insurance | No | 4485 (71.19) |
|  | Yes | 1815(28.81) |
| Family size | 1-3 | 2057 (32.7) |
|  | 4-6 | 3799 (60.3) |
|  | ≥7 | 444 (7.0) |
| Household children | 0 | 2037 (32.7) |
|  | 1-3 | 3892 (61.8) |
|  | ≥4 | 371 (5.9) |
| Household income (XAF)^Ɣ^ | < 50000 | 173 (2.7) |
|  | 50000 – 149999 | 1970 (31.3) |
|  | 150000 – 249999 | 1883(29.9) |
|  | 250000 – 499999 | 2047 (32.5) |
|  | ≥500000 | 227 (3.6) |
| Acute illnesses | No | 1358 (21.6) |
|  | Yes | 4942 (78.4) |
| Health expenditure (XAF)^Ɣ^ | None | 1355 (21.5) |
|  | < 50000 | 1767 (28.0) |
|  | 50000 – 149999 | 1866 (29.6) |
|  | 150000 – 249999 | 687 (10.9) |
|  | 250000 – 499999 | 448 (7.1) |
|  | ≥500000 | 177 (2.8) |

^Ѣ^Civil/traditional weeding or in concubinage

^ф^Own-account workers (not including administrative and professional workers), voluntary family workers, and employees/employers in establishments with less than 10 workers

^Ɣ^1 USD = 590.98 XAF

**Supplementary data S5:** Decomposition of the ordered logit regression model of the choice of the community-based health insurance

| Model | |  | IV | |  |  | V | |
| --- | --- | --- | --- | --- | --- | --- | --- | --- |
| Independent variables | |  | OR | SE |  |  | OR | SE |
| Age | |  |  |  |  |  |  |  |
|  | 18-24 |  | 1 |  |  |  | 1 |  |
|  | 25-44 |  | 0.27*** | 0.08 |  |  | 0.11*** | 0.03 |
|  | 45-64 |  | 0.29*** | 0.10 |  |  | 0.12*** | 0.04 |
|  | > 65 |  | 4.63** | 2.43 |  |  | 1.93 | 0.92 |
|  |  |  |  |  |  |  |  |  |
| Education level | |  |  |  |  |  |  |  |
|  | None |  | 1 |  |  |  | 1 |  |
|  | Primary |  | 5.97*** | 2.78 |  |  | 5.27*** | 2.45 |
|  | Secondary |  | 25.15*** | 11.27 |  |  | 22.4*** | 10.07 |
|  | Undergraduate |  | 4.95*** | 2.23 |  |  | 4.73** | 2.12 |
|  | Postgraduate |  | 8.51*** | 3.95 |  |  | 8.22*** | 3.79 |
|  |  |  |  |  |  |  |  |  |
| Working sector | |  |  |  |  |  |  |  |
|  | Public |  | 1 |  |  |  | 1 |  |
|  | Private |  | 0.35*** | 0.06 |  |  | 0.34*** | 0.06 |
|  | Informal |  | 11.36*** | 1.50 |  |  | 12.15*** | 1.55 |
|  | Retired |  | 1.06 | 0.55 |  |  | 1.29 | 0.66 |
|  |  |  |  |  |  |  |  |  |
| Household income (XAF)^Ɣ^ | |  |  |  |  |  |  |  |
|  | < 50000 |  | 1 |  |  |  | 1 |  |
|  | 50000 – 149999 |  | 160.56*** | 75.08 |  |  | 24.67*** | 8.57 |
|  | 150000 – 249999 |  | 6493.20*** | 3198.74 |  |  | 656.30*** | 240.19 |
|  | 250000 – 499999 |  | 19565.88*** | 9949.33 |  |  | 1854.20*** | 706.01 |
|  | ≥500000 |  | 54334.86*** | 31417.07 |  |  | 4469.00*** | 2068.82 |
|  |  |  |  |  |  |  |  |  |
| Household children | |  |  |  |  |  |  |  |
|  | 0 |  | 1 |  |  |  | 1 |  |
|  | 1-3 |  | 1.14 | 0.15 |  |  | 0.94 | 0.12 |
|  | ≥4 |  | 0.76 | 0.22 |  |  | 0.58 | 0.16 |
|  |  |  |  |  |  |  |  |  |
| Chronic illness | |  |  |  |  |  |  |  |
|  | No |  | 1 |  |  |  | 1 |  |
|  | Yes |  | 0.27*** | 0.03 |  |  | 0.28*** | 0.03 |
|  |  |  |  |  |  |  |  |  |
| Family size | |  |  |  |  |  |  |  |
|  | 1-3 |  | 1 |  |  |  | 1 |  |
|  | 4-6 |  | 0.19*** | 0.02 |  |  | 0.15*** | 0.02 |
|  | ≥7 |  | 0.03*** | 0.01 |  |  | 0.03*** | 0.01 |
|  |  |  |  |  |  |  |  |  |
| Health expenditure (XAF)^Ɣ^ | |  |  |  |  |  |  |  |
|  | None |  | 1 |  |  |  | 1 |  |
|  | < 50000 |  | 0.80 | 0.14 |  |  | 0.71* | 0.10 |
|  | 50000 – 149999 |  | 1.49* | 0.27 |  |  | 1.32 | 0.20 |
|  | 150000 – 249999 |  | 0.92 | 0.21 |  |  | 0.93 | 0.18 |
|  | 250000 – 499999 |  | 0.75 | 0.21 |  |  | 0.94 | 0.22 |
|  | ≥500000 |  | 0.45* | 0.17 |  |  | 0.57 | 0.19 |
|  |  |  |  |  |  |  |  |  |
| Family health status | |  |  |  |  |  |  |  |
|  | Bad |  | 1 |  |  |  |  |  |
|  | Passable |  | 0.57** | 0.10 |  |  |  |  |
|  | Good |  | 0.84 | 0.20 |  |  |  |  |
|  | |  |  |  |  |  |  |  |
| Health priority | |  |  |  |  |  |  |  |
|  | Low |  | 1 |  |  |  |  |  |
|  | Medium |  | 0.08*** | 0.05 |  |  |  |  |
|  | High |  | 0.08*** | 0.05 |  |  |  |  |
|  |  |  |  |  |  |  |  |  |
| Marital status | |  |  |  |  |  |  |  |
|  | Single |  | 1 |  |  |  |  |  |
|  | Married |  | 0.09*** | 0.02 |  |  |  |  |
|  | Divorced |  | 0.11*** | 0.03 |  |  |  |  |
|  | Widowed |  | 0.47* | 0.14 |  |  |  |  |
|  |  |  |  |  |  |  |  |  |
| Urban District | |  |  |  |  |  |  |  |
|  | Yaounde |  | 1 |  |  |  |  |  |
|  | Douala |  | 0.95 | 0.08 |  |  |  |  |
|  |  |  |  |  |  |  |  |  |
| Be aware of health insurance | |  |  |  |  |  |  |  |
|  | No |  | 1 |  |  |  |  |  |
|  | Yes |  | 1.00 | 0.11 |  |  |  |  |
|  |  |  |  |  |  |  |  |  |
|  | /cut |  | 3.99 | 0.63 |  |  | 5.52 | 0.53 |
|  |  |  |  |  |  |  |  |  |
|  |  | Prob > chi^2^ | 0.000 | |  |  | 0.000 | |
|  |  | Pseudo R^2^ | 0.54 | |  |  | 0.50 | |
|  |  | AIC | 3579.64 | |  |  | 3805.74 | |
|  |  | BIC | 3804.35 | |  |  | 3970.96 | |
|  |  | LR Test model I vs model II (LR chi^2^ = 224.09, *p* = 0.000) | | | | | | |

*, **, and *** indicate *p* < 0.05, *p* < 0.01, *p* < 0.001 significant levels, and non-significant, respectively (Z-test)

OR (Odd ratio), SE (standard error), AIC (Akaike's information criterion), and BIC (Bayesian information criterion)

Note: Voluntary community-based health insurance was select as the base outcome

^Ɣ^1 USD = 590.98 XAF

**Supplementary data S6:** Univariate analysis of premium for community-based health insurance

| Sociodemographic characteristics | | Scheme B | | |  | Scheme C | | |  | Prob > chi^2^ |
| --- | --- | --- | --- | --- | --- | --- | --- | --- | --- | --- |
|  |  | Mean (SD) | Median | N |  | Mean (SD) | Median | N |  |  |
|  |  |  |  |  |  |  |  |  |  |  |
| Gender | Male | 5323.56 (2747.48) | 5000.00 | 2063 |  | 4639.88 (2768.10) | 5000.00 | 1823 |  | *** |
|  | Female | 5361.08 (2485.30) | 5000.00 | 835 |  | 5271.71 (2721.51) | 5000.00 | 760 |  |  |
|  |  |  |  |  |  |  |  |  |  |  |
| Urban district | Yaounde | 5163.03 (2669.79) | 5000.00 | 1512 |  | 4684.52 (2747.06) | 5000.00 | 1260 |  | *** |
|  | Douala | 5521.28 (2667.44) | 5000.00 | 1386 |  | 4960.32 (2784.04) | 5000.00 | 1323 |  |  |
|  |  |  |  |  |  |  |  |  |  |  |
| Marital status | Single | 2610.74 (2019.20) | 2500.00 | 149 |  | 1805.84 (1459.55) | 1000.00 | 291 |  | *** |
|  | Married | 5572.24 (2594.78) | 5000.00 | 2464 |  | 5365.14 (2655.82) | 5000.00 | 1939 |  |  |
|  | Divorced | 5116.22 (2773.37) | 5000.00 | 185 |  | 4006.13 (2197.07) | 5000.00 | 163 |  |  |
|  | Widowed | 3935.00 (2594.34) | 2500.00 | 100 |  | 4650.00 (2715.79) | 5000.00 | 190 |  |  |
|  |  |  |  |  |  |  |  |  |  |  |
| Working sector | Public | 6541.74 (2538.01) | 7500.00 | 587 |  | 5491.58 (2605.37) | 5000.00 | 297 |  | *** |
|  | Private | 5851.78 (2487.64) | 5000.00 | 732 |  | 5500.00 (2717.84) | 5000.00 | 76 |  |  |
|  | Informal | 4648.64 (2588.75) | 5000.00 | 1544 |  | 4719.41 (2781.18) | 5000.00 | 2190 |  |  |
|  | Retired | 4514.29 (2855.56) | 2500.00 | 35 |  | 4025.00 (2268.06) | 5000.00 | 20 |  |  |
|  |  |  |  |  |  |  |  |  |  |  |
| Education level | None | 3607.14 (2527.05) | 2500.00 | 84 |  | 4821.43 (2493.12) | 5000.00 | 14 |  | *** |
|  | Primary | 4125.95 (2391.99) | 5000.00 | 262 |  | 4464.54 (2642.81) | 5000.00 | 141 |  |  |
|  | Secondary | 4360.10 (2281.88) | 5000.00 | 822 |  | 4004.82 (2638.40) | 2500.00 | 1141 |  |  |
|  | Undergraduate | 5841.05 (2637.79) | 5000.00 | 1403 |  | 5432.14 (2648.40) | 5000.00 | 980 |  |  |
|  | Postgraduate | 7021.41 (2451.90) | 7500.00 | 327 |  | 6107.49 (2731.71) | 7500.00 | 307 |  |  |
|  |  |  |  |  |  |  |  |  |  |  |
| Health priority | Low | 1214.29 (544.70) | 1000.00 | 14 |  | 1000.00 (0.00) | 1000.00 | 31 |  | *** |
|  | Medium | 4840.58 (2659.24) | 5000.00 | 207 |  | 3505.62 (2542.29) | 2500.00 | 178 |  |  |
|  | Hight | 5394.10 (2661.75) | 5000.00 | 2677 |  | 4974.73 (2741.57) | 5000.00 | 2374 |  |  |
|  |  |  |  |  |  |  |  |  |  |  |
| Chronic illness | No | 5258.03 (2728.48) | 5000.00 | 1494 |  | 4726.36 (2772.14) | 5000.00 | 1988 |  | *** |
|  | Yes | 5415.60 (2613.73) | 5000.00 | 1404 |  | 5157.98 (2734.51) | 5000.00 | 595 |  |  |
|  |  |  |  |  |  |  |  |  |  |  |
| Family health status | Bad | 5739.58 (2653.46) | 5000.00 | 672 |  | 5304.51 (2857.52) | 5000.00 | 266 |  | *** |
|  | Passable | 5161.66 (2640.33) | 5000.00 | 1896 |  | 5082.83 (2778.62) | 5000.00 | 1642 |  |  |
|  | Good | 5501.52 (2814.56) | 5000.00 | 330 |  | 4011.85 (2539.42) | 5000.00 | 675 |  |  |
|  |  |  |  |  |  |  |  |  |  |  |
| Age | 18-24 | 2801.37 (2343.34) | 1000.00 | 73 |  | 2103.60 (1903.49) | 1000.00 | 111 |  | *** |
|  | 25-44 | 5435.82 (2604.21) | 5000.00 | 2423 |  | 4496.17 (2709.16) | 5000.00 | 1695 |  |  |
|  | 45-64 | 5308.99 (2917.17) | 5000.00 | 356 |  | 6136.36 (2413.19) | 5000.00 | 715 |  |  |
|  | > 65 | 4206.52 (2680.33) | 2500.00 | 46 |  | 3596.77 (2800.52) | 2500.00 | 62 |  |  |
|  |  |  |  |  |  |  |  |  |  |  |
| Family size | 1-3 | 4742.42 (2891.74) | 5000.00 | 495 |  | 3550.67 (2493.18) | 2500.00 | 977 |  | *** |
|  | 4-6 | 5418.22 (2568.68) | 5000.00 | 2091 |  | 5541.42 (2624.51) | 5000.00 | 1509 |  |  |
|  | ≥7 | 5711.54 (2871.32) | 5000.00 | 312 |  | 6536.08 (2695.72) | 7500.00 | 97 |  |  |
|  |  |  |  |  |  |  |  |  |  |  |
| Be aware of health insurance | No | 4849.42 (2545.88) | 5000.00 | 2082 |  | 4633.81 (2676.50) | 5000.00 | 1973 |  | *** |
|  | Yes | 6571.69 (2595.55) | 7500.00 | 816 |  | 5446.72 (2967.26) | 5000.00 | 610 |  |  |
|  |  |  |  |  |  |  |  |  |  |  |
| Household children | 0 | 4701.01 (2721.09) | 5000.00 | 495 |  | 4159.24 (2631.71) | 5000.00 | 920 |  | *** |
|  | 1-3 | 5496.17 (2656.26) | 5000.00 | 2091 |  | 5154.75 (2767.43) | 5000.00 | 1622 |  |  |
|  | ≥4 | 5254.81 (2572.42) | 5000.00 | 312 |  | 6768.29 (2636.52) | 7500.00 | 41 |  |  |
|  |  |  |  |  |  |  |  |  |  |  |
| Household income (XAF)^Ɣ^ | < 50000 | 1395.35 (663.43) | 1000.00 | 129 |  | 1000.00 (0.00) | 1000.00 | 26 |  | *** |
|  | 50000 – 149999 | 3797.99 (1667.98) | 5000.00 | 1542 |  | 1136.08 (466.83) | 1000.00 | 316 |  |  |
|  | 150000 – 249999 | 6721.56 (1522.06) | 7500.00 | 668 |  | 3505.95 (1531.41) | 2500.00 | 1009 |  |  |
|  | 250000 – 499999 | 8764.59 (1415.60) | 10000.00 | 514 |  | 6835.94 (2074.73) | 7500.00 | 1152 |  |  |
|  | ≥500000 | 9500.00 (1011.30) | 10000.00 | 45 |  | 8343.75 (2026.09) | 10000.00 | 80 |  |  |
|  |  |  |  |  |  |  |  |  |  |  |
| Health expenditure (XAF)^Ɣ^ | None | 4633.01 (2548.39) | 5000.00 | 312 |  | 4019.74 (2579.63) | 2500.00 | 532 |  | *** |
|  | < 50000 | 5283.22 (2653.20) | 5000.00 | 888 |  | 4928.97 (2849.77) | 5000.00 | 718 |  |  |
|  | 50000 – 149999 | 5196.16 (2639.22) | 5000.00 | 808 |  | 4958.97 (2751.84) | 5000.00 | 987 |  |  |
|  | 150000 – 249999 | 5513.72 (2830.71) | 5000.00 | 401 |  | 5381.05 (2756.84) | 5000.00 | 248 |  |  |
|  | 250000 – 499999 | 5722.70 (2739.13) | 5000.00 | 348 |  | 5572.37 (2476.16) | 5000.00 | 76 |  |  |
|  | ≥500000 | 6531.91 (2044.34) | 7500.00 | 141 |  | 6136.36 (2279.21) | 5000.00 | 22 |  |  |
|  |  |  |  |  |  |  |  |  |  |  |
| Overall | - | 5334.37 (2674.20) | 5000.00 | 2898 |  | 4825.78 (2768.96) | 5000.00 | 2583 |  | - |

*, **, and *** indicate *p* < 0.05, *p* < 0.01, *p* < 0.001 significant levels, and non-significant, respectively (Chi^2^ test)

B (Compulsory scheme) and C (voluntary scheme)

^Ɣ^1 USD = 590.98 XAF

**Supplementary data S7:** Decomposition of the ordinary least squares and generalized linear model of the premium of community-based health insurance

| Model | |  | Scheme B | | | | |  | Scheme C | | | | |
| --- | --- | --- | --- | --- | --- | --- | --- | --- | --- | --- | --- | --- | --- |
|  |  |  | OLS | |  | GLM | |  | OLS | |  | GLM | |
| Independent variables | |  | Coef. | SE |  | Coef. | SE |  | Coef. | SE |  | Coef. | SE |
| Age |  |  |  |  |  |  |  |  |  |  |  |  |  |
|  | 25-44 |  | 0.16 | 0.09 |  | 0.07* | 0.03 |  | 0.17 | 0.09 |  | 0.09** | 0.03 |
|  | 45-64 |  | 0.06 | 0.10 |  | 0.04 | 0.04 |  | 0.30** | 0.09 |  | 0.15*** | 0.03 |
|  | > 65 |  | -0.01 | 0.15 |  | -0.01 | 0.06 |  | 0.18 | 0.14 |  | 0.03 | 0.05 |
|  |  |  |  |  |  |  |  |  |  |  |  |  |  |
| Education level | |  |  |  |  |  |  |  |  |  |  |  |  |
|  | Primary |  | 0.04 | 0.09 |  | 0.01 | 0.03 |  | -0.41* | 0.20 |  | -0.19** | 0.07 |
|  | Secondary |  | 0.00 | 0.08 |  | 0.00 | 0.03 |  | -0.19 | 0.20 |  | -0.09 | 0.07 |
|  | Undergraduate |  | -0.05 | 0.08 |  | -0.01 | 0.03 |  | -0.07 | 0.20 |  | -0.06 | 0.07 |
|  | Postgraduate |  | -0.04 | 0.09 |  | -0.01 | 0.03 |  | 0.05 | 0.21 |  | -0.03 | 0.07 |
|  |  |  |  |  |  |  |  |  |  |  |  |  |  |
| Working sector | |  |  |  |  |  |  |  |  |  |  |  |  |
|  | Private |  | -0.01 | 0.04 |  | 0.00 | 0.01 |  | 0.05 | 0.09 |  | 0.02 | 0.03 |
|  | Informal |  | 0.04 | 0.04 |  | 0.01 | 0.01 |  | -0.10 | 0.05 |  | -0.05** | 0.02 |
|  | Retired |  | 0.14 | 0.14 |  | 0.04 | 0.05 |  | -0.15 | 0.18 |  | 0.02 | 0.06 |
|  |  |  |  |  |  |  |  |  |  |  |  |  |  |
| Household children | |  |  |  |  |  |  |  |  |  |  |  |  |
|  | 1-3 |  | 0.02 | 0.04 |  | 0.00 | 0.02 |  | 0.15*** | 0.04 |  | 0.05*** | 0.01 |
|  | ≥4 |  | -0.01 | 0.06 |  | 0.00 | 0.02 |  | 0.47*** | 0.13 |  | 0.13** | 0.04 |
|  |  |  |  |  |  |  |  |  |  |  |  |  |  |
| Chronic illness | |  |  |  |  |  |  |  |  |  |  |  |  |
|  | Yes |  | -0.04 | 0.03 |  | -0.01 | 0.01 |  | -0.17*** | 0.04 |  | -0.06*** | 0.02 |
|  |  |  |  |  |  |  |  |  |  |  |  |  |  |
| Household income (XAF)^Ɣ^ | |  |  |  |  |  |  |  |  |  |  |  |  |
|  | 50000 – 149999 |  | 1.20*** | 0.07 |  | 0.66*** | 0.03 |  | 0.00 | 0.18 |  | 0.02 | 0.06 |
|  | 150000 – 249999 |  | 2.40*** | 0.08 |  | 1.05*** | 0.03 |  | 1.22*** | 0.19 |  | 0.77*** | 0.07 |
|  | 250000 – 499999 |  | 3.25*** | 0.08 |  | 1.26*** | 0.03 |  | 2.54*** | 0.19 |  | 1.21*** | 0.07 |
|  | ≥500000 |  | 3.61*** | 0.13 |  | 1.35*** | 0.05 |  | 2.96*** | 0.21 |  | 1.30*** | 0.07 |
|  | |  |  |  |  |  |  |  |  |  |  |  |  |
| Health expenditure (XAF)^Ɣ^ | |  |  |  |  |  |  |  |  |  |  |  |  |
|  | < 50000 |  | 0.02 | 0.05 |  | 0.01 | 0.02 |  | 0.06 | 0.05 |  | 0.04* | 0.02 |
|  | 50000 – 149999 |  | 0.01 | 0.06 |  | 0.00 | 0.02 |  | 0.01 | 0.05 |  | 0.01 | 0.02 |
|  | 150000 – 249999 |  | 0.03 | 0.07 |  | 0.00 | 0.02 |  | -0.22** | 0.08 |  | -0.05* | 0.03 |
|  | 250000 – 499999 |  | 0.12 | 0.08 |  | 0.04 | 0.03 |  | -0.08 | 0.12 |  | 0.02 | 0.04 |
|  | ≥500000 |  | 0.25** | 0.09 |  | 0.09** | 0.03 |  | 0.30 | 0.17 |  | 0.14* | 0.06 |
|  |  |  |  |  |  |  |  |  |  |  |  |  |  |
| Family health status | |  |  |  |  |  |  |  |  |  |  |  |  |
|  | Passable |  | 0.04 | 0.05 |  | 0.02 | 0.02 |  | -0.11 | 0.07 |  | -0.02 | 0.03 |
|  | Good |  | 0.12 | 0.06 |  | 0.03 | 0.02 |  | -0.18* | 0.09 |  | -0.03 | 0.03 |
|  |  |  |  |  |  |  |  |  |  |  |  |  |  |
| Health priority | |  |  |  |  |  |  |  |  |  |  |  |  |
|  | Medium |  | 0.12 | 0.20 |  | 0.10 | 0.07 |  | -0.12 | 0.18 |  | -0.03 | 0.06 |
|  | Hight |  | 0.11 | 0.20 |  | 0.08 | 0.08 |  | -0.23 | 0.18 |  | -0.06 | 0.06 |
|  |  |  |  |  |  |  |  |  |  |  |  |  |  |
| Urban District | |  |  |  |  |  |  |  |  |  |  |  |  |
|  | Douala |  | 0.18*** | 0.03 |  | 0.06*** | 0.01 |  | 0.14*** | 0.03 |  | 0.06*** | 0.01 |
|  |  |  |  |  |  |  |  |  |  |  |  |  |  |
| Be aware of health insurance | | | |  |  |  |  |  |  |  |  |  |  |
|  | Yes |  | 0.02 | 0.03 |  | 0.01 | 0.01 |  | -0.07 | 0.04 |  | -0.04** | 0.01 |
|  |  |  |  |  |  |  |  |  |  |  |  |  |  |
| _cons | |  | 0.86*** | 0.19 |  | 0.05 | 0.07 |  | 1.41*** | 0.27 |  | 0.14 | 0.10 |
|  |  |  |  |  |  |  |  |  |  |  |  |  |  |
|  |  | AIC | 5776.75 | |  | 12152.26 | |  | 5461.23 | |  | 10285.72 | |
|  |  | BIC | 5949.93 | |  | 12325.44 | |  | 5631.08 | |  | 10455.56 | |

*, **, and *** indicate *p* < 0.05, *p* < 0.01, *p* < 0.001 significant levels, and non-significant, respectively (Z-test)

B (Compulsory scheme) and C (voluntary scheme), OLS (Ordinary least square model), GLM (generalized linear model), OR (relative risk ratios), SE (standard error), AIC (Akaike's information criterion), and BIC (Bayesian information criterion)

^Ɣ^1 USD = 590.98 XAF
